# Advances in the treatment of solid tumors in children and adolescents

**DOI:** 10.1002/cai2.66

**Published:** 2023-04-08

**Authors:** Jing Tian, Jiayu Wang, Sidan Li

**Affiliations:** ^1^ Beijing Key Laboratory of Pediatric Hematology Oncology, Key Laboratory of Major Diseases in Children, National Key Discipline of Pediatrics (Capital Medical University), Ministry of Education, Beijing Children's Hospital, Hematology Center, National Center for Children's Health Capital Medical University Beijing China; ^2^ Department of Medical Oncology, National Cancer Center, National Clinical Research Center for Cancer, Cancer Hospital Chinese Academy of Medical Sciences and Peking Union Medical College Beijing China

**Keywords:** children and adolescents, immune checkpoint inhibitors, treatment, tumor

## Abstract

Tumor is one of the leading causes of death in children (0 to 14‐year‐old) and adolescents (15 to 19‐year‐old) worldwide. Unlike adult tumors, childhood and adolescent tumors are unique in their type, molecular characteristics, and pathogenesis, and their treatment involves many challenges. In recent years, with the development of a large number of clinical studies, the survival rate of children and adolescents with tumors has improved significantly. The extensive research and application of optimized treatment regimens and new targeted drugs have led to new hope for the treatment of childhood and adolescent tumors. This article reviews the clinical and basic research and treatment of childhood and adolescent tumors and provides new ideas for the future development of precise treatment of childhood and adolescent tumors.

## Graphical abstract

The tumor is one of the threats to children and adolescents' life and health, causing death and reduced quality of life. But there are few studies on them. Improving the survival rate and survival time of patients is our common goal. We reviewed the literature in recent years, and summarized the new progress achieved in terms of chemotherapy regimens, molecular targeted drugs, and immunotherapy, to provide new ideas for the treatment of tumors in children and adolescents.

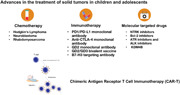

**Abbreviations**
ADC: antibody–drug conjugate
ADCC: antibody‐dependent cell‐mediated cytotoxicity
ALL: acute lymphoblastic leukemia
ALT: alanine aminotransferase
AMT: adrenergic‐to‐mesenchymal transition
aRMS: alveolar RMS
AST: aspartate aminotransferase
CTLA‐4: cytotoxic T lymphocyte antigen 4
DLBCL: diffuse large B‐cell lymphoma
EFS: event‐free survival
FL: follicular lymphoma
HL: Hodgkin's Lymphoma
LBL: lymphoblastic lymphoma
NB: neuroblastoma
NCPCS: National Center for Pediatric Cancer Surveillance
NHL: non‐Hodgkin's lymphoma
NTRK: neurotrophic receptor tyrosine kinase
ORR: overall response rate
OS: overall survival
PFS: progression‐free survival
RMS: rhabdomyosarcoma
RP2D: recommended phase II dose
TAA: tumor‐associated antigen

## 1 INTRODUCTION

Tumor is one of the main causes of death in children and adolescents, seriously endangering health. Tumors in children and adolescents tend to present earlier than in adults, are usually caused by maturation arrest in developing immature cell types, are of unknown etiology, and are often driven by mutations in a single gene [1]. Because tumor types and biological characteristics in children and adolescents are different from adult tumors, some adult treatment options are not suitable for children, and clinical trials are lacking, which has placed a huge burden on families and society. A cross‐sectional study conducted by the National Center for Pediatric Cancer Surveillance (NCPCS) [2] showed that the incidence of cancer in China was 122.86/million for children and 137.64/million for adolescents, among which leukemia accounted for about 32% of childhood tumors, lymphoma accounted for about 9.7%, and the remaining nearly 60% of tumors are solid tumors. In childhood tumors, patients are in the stage of rapid development, and the life expectancy after tumor treatment is longer. Therefore, attention should be paid to the long‐term effects caused by tumor types and treatment methods at the same time [3]. As more clinical studies are conducted, treatment for tumors in children and adolescents become more precise.

## 2 NEW DEVELOPMENTS IN CHEMOTHERAPY REGIMENS

### 2.1 Hodgkin's lymphoma (HL)

Chemotherapy regimens such as MOPP (mechlorethamine, vincristine, procarbazine, and prednisone) and ABVD (doxorubicin, bleomycin, vinblastine, and dacarbazine) are clinically approved chemotherapy regimens for children with HL. Based on the consideration of short‐ and long‐term toxic side effects in children, clinical adjustments based on MOPP and ABVD that have been made in a variety of treatment regimens suitable for children and adolescents, including OEPA (vincristine, etoposide, prednisone, and doxorubicin) induction therapy, COPP (cyclophosphamide, vincristine, procarbazine, and prednisone) intensive therapy, and radiotherapy, can lead to good survival rates and high disease control rates. However, long‐term treatment affects survival and quality of life. The goal of optimization is to improve the early disease control rate, reduce the disease recurrence rate, and reduce long‐term adverse reactions.

A phase III clinical trial named AHOD1331 [4] compared the effectiveness and safety of brentuximab vedotin combined with chemotherapy AVE‐PC (doxorubicin, vincristine, etoposide, prednisone, and cyclophosphamide) in high‐risk children and adolescents with HL, compared with standard chemotherapy ABVE‐PC (doxorubicin, bleomycin, vincristine, etoposide, prednisone, and cyclophosphamide). It showed that brentuximab vedotin significantly reduced the risk of disease progression, recurrence, secondary tumors, and death, with a 3‐year event‐free survival (EFS) of 92.1% versus 82.5%. The FDA officially approved brentuximab vedotin for the first‐line treatment of high‐risk HL patients over 2‐year‐old, and it has become a new standard treatment. In a European noninferiority randomized‐controlled clinical trial [5] in the middle and advanced stages of juvenile HL patients, patients with partial remission or better response to chemotherapy are considered to have sufficient early response, and then radiotherapy in later treatment is withdrawn. The study shows that the 5‐year EFS in the radiotherapy‐free group was 90.1%. At the same time, in the intensive treatment phase, the COPDAC (cyclophosphamide, vincristine, dacarbazine, and prednisone) chemotherapy regimen that replaced procarbazine with dacarbazine, although slightly reduced in effectiveness, greatly reduced reproductive system toxicity. This treatment regimen may change the standard of care for HL.

### 2.2 Neuroblastoma (NB)

NB treatment is a multidisciplinary comprehensive treatment based on surgery and chemotherapy. The three stages of treatment include induction therapy (chemotherapy and surgery), intensive therapy (high‐dose chemotherapy plus autologous stem cell transplantation and radiotherapy), and maintenance therapy (isotretinoin and GD2 monoclonal antibody).

The European rapid COJEC (rCOJEC) regimen consists of cisplatin, vincristine, carboplatin, etoposide, and cyclophosphamide, while the US Memorial Sloan Kettering Cancer Center regimen (MSKCC‐N5) uses high‐dose cyclophosphamide, a combination of doxorubicin and vincristine alternating with cisplatin and etoposide. The study of the SIOPEN collaborative group [6] compared the treatment effects of the two, and there was no significant difference in the complete remission rate of metastases, overall remission rate, 3‐year EFS, and overall survival (OS), but the European scheme led to fewer side effects.

### 2.3 Rhabdomyosarcoma (RMS)

RMS is the most common soft tissue tumor in children. Multidisciplinary treatment is recommended. Surgical treatment is the first choice for patients whose tumors can be completely resected after evaluation. Chemotherapy is administered after clear pathology and risk grouping. Children in the low‐risk group received VA (vincristine and dactinomycin) and VAC (vincristine, dactinomycin, and cyclophosphamide) regimens, children in the intermediate‐risk group received VAC (same as the low‐risk group) and VI (vincristine and irinotecan) regimens, and children in the high‐risk group received VAC, VI regimens (same as the intermediate‐risk group) and VDC (vincristine, adriamycin, and cyclophosphamide)/IE (ifosfamide and etoposide) regimens. The IVA (ifosfamide, vincristine, and dactinomycin) chemotherapy regimen is still recommended as the first‐line chemotherapy for children with high‐risk nonmetastatic RMS. In a phase 3 clinical trial, the efficacy of early dose intensification with doxorubicin added to the IVA regimen was investigated in children with local RMS. There was no significant difference in 3‐year EFS (IVA 67.5% vs. IVA plus doxorubicin 63.3%, *p* = 0.33), and two treatment‐related deaths were reported in the IVA plus doxorubicin group [7].

In preclinical studies, Temsirolimus showed promising antitumor activity either as a single agent or in combination with chemotherapy. The safety and efficacy of Vnblastine combined with Temsirolimus in the treatment of relapsed and refractory pediatric solid tumors have been verified in clinical trials [8]. Recent studies have shown that compared with chemotherapy combined with Bevacizumab, chemotherapy combined with Temsirolimus can lead to better EFS in children with RMS (69.1% vs. 54.6%) [9].

Vincristine combined with irinotecan can achieve an overall response rate (ORR) of 70% in the treatment of newly diagnosed metastatic RMS, but VI therapy is still not effective for relapsed or refractory RMS. The European RMS Research Group designed a phase II clinical trial of the VI regimen combined with temozolomide (T) in the treatment of relapsed or refractory RMS [10]. Studies have shown that this regimen has acceptable safety, and the ORR has increased from 31% with the combination of two drugs (VI) to 44% with the combination of three drugs (VIT), which has significantly improved the progression‐free survival (PFS) and OS of patients, and has the potential to become a new therapy for relapsed or refractory RMS.

## 3 CLINICAL RESEARCH OF NEW DRUGS

### 3.1 Immunotherapy

#### 3.1.1 PD1/PD‐L1 monoclonal antibody

T cells recognize tumor cell‐specific antigens and activate the immune response. PD‐L1 is expressed on the surface of normal cells, preventing T cells from being activated to attack their own normal tissue cells. However, a large number of PD‐L1 molecules are expressed on the surface of tumor cells, and due to the combination of PD1 and PD‐L1, T cells are unable to recognize and kill tumor cells. The discovery of this immune evasion mechanism of tumor cells has become a new target for tumor therapy. Tumors in children have a low mutation rate, but they can respond to PD‐1 inhibitors, so as long as there is enough antigen load to drive T cell activation, the ideal therapeutic effect on tumors in children can be achieved [11]. Approximately 9% of childhood tumors are positive for PD‐L1, with Burkitt lymphoma, glioblastoma, and NB being the most common [12].

Immunotherapy with PD‐1 inhibitors has shown success in some childhood tumors. Nivolumab is a humanized IgG4 monoclonal PD‐1 antibody approved for use in metastatic colon cancer with high microsatellite instability or mismatch repair deficiency. It can be used as a second‐line treatment for advanced melanoma, nonsmall cell lung cancer, and relapsed/refractory HL. In a clinical study exploring the safety and effectiveness of nivolumab in the treatment of tumors in children and adolescents [13], responses were observed in some patients of lymphoma, and 3 mg/kg every 2 weeks was an acceptable safe and effective dose for patients. Low‐dose cyclophosphamide chemotherapy can eliminate regulatory T cells and play an immunomodulatory role in NK cells. Therefore, a study evaluated the safety and efficacy of the PD‐1 inhibitor nivolumab combined with low‐dose cyclophosphamide in the treatment of some children with relapsed/refractory tumors. The results showed that it was well tolerated, but the addition of cyclophosphamide could not compensate for the infiltration of tumor T cells and regulate the immunosuppression of the tumor microenvironment, and the drug effect was limited [14].

Pembrolizumab can bind to PD‐1 and prevent its interaction with PD‐L1. Currently, pembrolizumab has been approved for the treatment of advanced tumors in adults, such as melanoma and nonsmall cell lung cancer. In a phase I–II study of pembrolizumab in the treatment of children and adolescents with advanced melanoma or advanced relapsed/refractory solid tumors or lymphoma [15], all 15 children with relapsed/refractory HL showed good tolerance and response, with an objective response rate of 60%, but the objective response rate in children with other solid tumors was 5.9%, suggesting that pembrolizumab is not effective for most children with solid tumors. In the two clinical trials of ADVL1412 and KEYNOTE‐051, no good objective response was observed in patients with nonlymphoma solid tumors (ADVL1412 was 0, KEYNOTE‐051 was 5.9%), considered to be associated with the low mutation rate and the low PD‐L1 positive rate of childhood tumors. In ADVL1412 [13], the results of PD‐L1 positive detection (based on PD‐L1 expression >1%) showed that only 14% of nonlymphoma patients were PD‐L1 positive, and 90% of lymphoma patients were PD‐L1 positive. Therefore, the efficacy of PD1/PD‐L1 monoclonal antibody monotherapy in the treatment of children with solid tumors is not good, and further research focused on the efficacy and safety of its combination with chemotherapy in the treatment of children with nonlymphoma solid tumors is needed.

Atezolizumab can bind to PD‐L1 on tumor cells and block its interaction with PD‐1 on T cells and antigen‐presenting cells, relieve immunosuppression, and promote T cells to attack tumor cells. The iMATRIX clinical trial [16] explored the efficacy and safety of atezolizumab in the treatment of relapsed/refractory solid tumors in children and adolescents. Out of 87 evaluable tumor patients, only 4 cases showed objective response (1 patient with malignant rhabdoidcancer, 1 patient with non‐Hodgkin's lymphoma (NHL), 2 patients with HL).

#### 3.1.2 Anti‐CTLA‐4 monoclonal antibody

Cytotoxic T lymphocyte antigen 4 (CTLA‐4), an immune protein expressed on the surface of activated T cells, is responsible for transmitting inhibitory signals. The mechanism of CTLA‐4 mediating the suppressive function of T cells may be to compete with the T cell activator CD28 to bind B7 molecules on the surface of antigen‐presenting cells, resulting in the inhibition of T cell activation. Ipilimumab is a fully human monoclonal antibody that blocks the interaction between CTLA‐4 and B7 molecules, allowing B7‐1 molecules to preferentially bind to CD28 and activate T cells [17]. In a phase I clinical trial of ipilimumab in the treatment of advanced solid tumors in children [18], it was determined that the tolerable dose for children was 3 mg/kg, which was the same as that for adults, but the efficacy of ipilimumab monotherapy in the treatment of childhood tumors was not observed. Responsiveness was observed in some patients in a phase II trial of ipilimumab in adolescents with advanced melanoma [19]. In October 2022, the researchers of the phase II ADVL1412 trial [20] continued to present the results of exploring the dose response of nivolumab combined with ipilimumab in the treatment of children with relapsed/refractory solid tumors, the result showed that 3 mg/kg nivolumab and 1 mg/kg ipilimumab were defined as recommended phase II dose (RP2D). On further exploration of drug toxicity and adverse reactions, it was found that two of the eight patients maintained their partial remission status until 36 months after the end of follow‐up, suggesting that the combination of the two drugs has expected clinical effects. When the curative effect of the anti‐CTLA‐4 monoclonal antibody is not satisfactory, combining with other immunosuppressants to synergistically activate T cells and improve the antitumor efficacy and the survival rate of tumor patients is an avenue worthy of further exploration in the future.

#### 3.1.3 GD2 monoclonal antibody

Monoclonal antibodies targeting GD2 are part of the standard of care for high‐risk NB patients, generally in follow‐up therapy. St Jude Children's Hospital in the United States launched a new clinical trial [21], they tried to treat 64 cases of newly diagnosed high‐risk NB with the GD2 monoclonal antibody in combination with chemotherapy by extending the use of the GD2 monoclonal antibody (hu14.18K322A) to the induction and intensive treatment phase, and there was a significant improvement in the early response of patients, with a 3‐year EFS of 73.7% and OS of 86%. Naxitamab is a humanized antiganglioside disialoganglioside (GD2) monoclonal antibody, Naxitamab combined with GM‐CSF showed good efficacy in the consolidation treatment of high‐risk neuroblastoma patients, with a 3‐year OS rate of 82.4% [22].

The antitumor mechanism of the GD2 monoclonal antibody involves antibody‐dependent cell‐mediated cytotoxicity (ADCC). The cytokine IL‐2 can enhance the ADCC effect. It was initially part of the NB immunotherapy regimen, but uncertainty of its high‐dose‐related toxicity and low‐dose efficacy called into question its therapeutic benefit. To improve the antitumor efficacy, attention has been focused on IL‐15 and IL‐21, which are in the same family as IL‐2. The researchers linked IL‐15 and IL‐21 to the anti‐GD2 antibody hu14.18 and found that the two‐novel antibody–cytokine conjugates, hu14.18‐IL15 and ‐IL21, were able to induce complete regression and prolong survival in NB tumor models, which had a stronger ADCC effect than hu14.18‐IL2 [23]. This study has important implications for NB clinical trials and other preclinical models of childhood solid tumors expressing GD2.

Poor effects of GD2 also occur in some patients. A study found that adrenergic‐to‐mesenchymal transition (AMT)‐mediated transcriptional recombination led to the ineffectiveness of anti‐GD2 antibodies by downregulation of sialyltransferase GD3 synthetase (GD3S; ST8SIA1), while drug inhibition of H3K27me3 histone methyltransferase EZH2 restored its expression and restored sensitivity to GD2 antibodies, suggesting that the combination of EZH2 inhibitors and anti‐GD2 antibodies in clinical trials can help improve the prognosis of children with NB [24].

A new treatment method, the GD2 monoclonal antibody vaccine, is used to treat tumors. The relevant antigens are injected into the human body to continuously induce antibody production and promote the generation of memory B lymphocyte cells, which can quickly take effect when the disease relapses. Antitumor vaccines can also reduce autoimmune epitopes as much as possible through cleavage, tolerance, and other mechanisms to avoid attacking normal tissues.

#### 3.1.4 GD2/GD3 bivalent vaccine

The team of Brian H. Kushner [25] in the United States developed a GD2/GD3 bivalent vaccine, which can induce a durable and effective antibody response in high‐risk NB patients with disease progression, and it has good safety. In patients with high‐risk neuroblastoma with disease progression, the GD2/GD3 bivalent vaccine combined with β‐glucan produced high titers of anti‐GD2‐IgG and improved patient survival [26].

#### 3.1.5 B7‐H3 targeting antibody

B7‐H3 protein is a type I transmembrane protein encoded by the CD276 gene. It is an immune checkpoint belonging to the same protein family as PD‐L1. The expression level is low in normal human tissues, but the expression level is very high in most childhood solid tumors, especially in osteosarcoma and nephroblastoma, and its high expression may be related to tumor progression and metastasis. The m276‐SL‐PBD antibody–drug conjugate (ADC) developed by a research team [27] targeting B7‐H3 has shown strong antitumor effects in mice models of various childhood tumor cell and is a brand‐new pediatric solid tumor therapeutic target.

### 3.2 Molecular targeted drugs

#### 3.2.1 Neurotrophic receptor tyrosine kinase (NTRK) inhibitors

The NTRK fusion gene has been found in some adult and childhood malignant tumors, and the expression of abnormal oncoprotein activates downstream cancer‐promoting pathways. The ETV6‐NTRK3 fusion gene has been found in children's fibrosarcoma tissue. Larotrectinib is an NTRK inhibitor, which can prevent the fusion of NTRK and other genes, inhibit cell signal transduction, inhibit the growth and proliferation of tumor cells, and promote cell apoptosis. In a summary of 3 phase I/II clinical trials of larotrectinib [28], objective response was observed in 68% of patients with NTRK gene fusion tumors. Larotrectinib at 100 mg/m^2^ twice a day has been confirmed to be a safe and effective dose that can be tolerated by children. The most common adverse reactions were an increase in the levels of aspartate aminotransferase (AST) and alanine aminotransferase (ALT), and nausea.

#### 3.2.2 Bcl‐2 inhibitors

The Bcl‐2 gene can inhibit and resist cells apoptosis. It is an oncogene with clear carcinogenesis. Bcl‐2 translocation can be seen in 80%–90% of follicular lymphoma (FL) patients, and t(14;18)(q32;q21)/IgH‐BCL‐2 is the characteristic translocation of FL [29]. Bcl‐2 rearrangement and translocation also exist in 15%–20% of diffuse large B‐cell lymphoma (DLBCL) patients [30]. The study showed that the expression level of Bcl‐2 is inversely proportional to the survival rate of patients, and the prognosis is poor [31, 32]. Also, overexpression of Bcl‐2 in NHL patients increases resistance to common chemotherapy regimens such as R‐CHOP [33]. In children with FL, although Bcl‐2 protein expression can be detected, t(14;18)(q32;q21) can only be detected in rare cases [34], which is different from adult FL. Bcl‐2 protein expression in children with DLBCL can be used as an immunohistochemical feature for diagnosis [35].

Venetoclax (ABT‐199) selectively inhibits the antiapoptotic protein Bcl‐2. Clinical data suggest that venetoclax monotherapy can effectively kill NHL cells [36]. Venetoclax was used for treatment in adult R/R NHL phase I clinical trials and it was found to be well tolerated, with an overall response rate of 44%, but the efficacy varied with the histological subtype of lymphoma [37].

Special attention should be paid to the fact that venetoclax cannot inhibit the expression of two key drug resistance genes BCL‐XL and MCL‐1 [38], so it is recommended to combine it with rituximab and chemotherapy to improve the efficacy. In a study of venetoclax in the treatment of children and adolescents with acute lymphoblastic leukemia (ALL) and lymphoblastic lymphoma (LBL) [39], the safety and efficacy of ABT‐199 in this study were evaluated; 61% of children achieved complete remission. In addition, in the phase I trial [40] of venetoclax combined with low‐dose navitoclax and chemotherapy, it was found to be well tolerated and effective in the treatment of ALL and LBL in children and adults. This combination can simultaneously inhibit Bcl‐2 and Bcl‐XL, to make up for the deficiency of venetoclax monotherapy.

#### 3.2.3 ATR inhibitors and ALK inhibitors

High‐risk NB is often accompanied by amplification of the oncogene MYCN and gene mutation of ALK. Basic studies have shown that the ATR inhibitor (BAY1895344) combined with the ALK inhibitor (lorlatinib) can lead to complete regression of tumor cells in an ALK‐driven NB mouse model [41]. Crizotinib has been approved for patients with EML4‐ALK‐positive nonsmall cell lung cancer. ALK is highly expressed in pediatric rhabdomyosarcoma, and high sensitivity of RMS to the ALK inhibitor has been found in vitro. However, ALK inhibitors are ineffective in vivo because the PDX signaling pathway expressing ALK in vivo is not affected by ALK inhibitors [42]. In a phase II trial of Alectinib in ALK‐positive anaplastic large B‐cell lymphoma, the efficacy and safety of Alectinib in patients older than 6 years were explored, and the 1‐year OS rate reached 70%. The most common adverse event was neutropenia, which provided a reliable basis for clinical treatment [43]. Entrectinib is a TRKA/B/C, ROS1, and ALK inhibitor. In a clinical trial of the recommended phase 2 dose of Entrectinib, the ORR of children and young adults with solid or primary CNS tumors harboring NTRK, ROS1, or ALK gene fusion was 57.7%, entrectinib in this study achieved ideal clinical efficacy [44]. At present, Entrectinib has been officially approved by the FDA for patients with gene fusion tumors.

#### 3.2.4 KDM4B

Childhood RMS is the most common soft tissue sarcoma in children. The fusion gene PAX‐FOXO1 is a chimeric transcription factor and a known oncogenic driver, and the transformation of alveolar RMS(aRMS) is partially driven by the PAX3‐FOXO1 or its variant PAX7‐FOXO1 fusion protein. PAX3‐FOXO1 fusion‐positive aRMS was more aggressive than fusion‐negative RMS, had a higher rate of metastasis and a worse prognosis. Studies have shown that inhibition of histone lysine demethylase 4B (KDM4B) can delay the growth of tumor cells and inhibit the core transcription factor PAX3‐FOXO1, suggesting that KDM4B may be a potential therapeutic target for malignant childhood tumors such as PAX‐FOXO1+ RMS [45].

### 3.3 Chimeric antigen receptor T cell immunotherapy (CAR‐T)

CAR‐T therapy is a new type of precise targeted therapy in recent years. T cells are isolated and extracted in vitro, and T cells are genetically modified to carry chimeric antigen receptors. The CAR‐T cells are massively expanded in vitro and infused back into the patient's body to recognize tumor cells, activate immune response, and kill tumor cells. However, due to the diverse expression of tumor‐associated antigen (TAA), CAR‐T cells cannot specifically recognize tumor cells, resulting in poor efficacy. At present, CD19 CAR‐T has been approved by the United States for the treatment of patients with acute B‐lymphoblastic leukemia and DLBCL. At present, CAR‐T therapies targeting GD2, L1‐CAM, and CD276 and other tumor antigens have entered clinical trials. However, there are few studies on evaluation of CAR‐T efficacy for children with solid tumors.

NB is a common extracranial solid tumor in children. Overexpression of GPC2 and CD276 can be detected in NB tissue. GPC2 is essential for the growth and differentiation of neurons. CD276 is involved in tumor immune evasion and tumor blood vessel formation. Their overexpression is associated with poor prognosis in tumor patients. Therefore, CAR‐T therapy targeting GPC2 and CD276 has led to new opportunities for NB patients. In a study published in August 2022, the team led by Javed Khan [46] used BiCisCAR to target multiple TAAs and overcome the heterogeneous expression of target antigens in solid tumors and identified an effective CAR‐T therapy for NB. In this study, 95% of NB samples had high expression of GPC2 and CD276, and CT3 and MGB7H3‐LH were confirmed to be effective CAR constructs for recognizing GPC2‐ and CD276‐expressing tumor cells using multimodal single‐cell analysis methods and cytotoxicity assays. GPC2/CD276 BiCisCAR showed superior antitumor activity in vivo in animal testing, in which 33.3% of mice showed good tumor suppression or even tumor elimination activity. The researchers measured the proportion of CAR‐T cells in models expressing GPC2 or CD276 after 21 days of infusion of CAR‐T cells and found that CAR‐T cells were persistent and reduced exhaustion.

### 3.4 Other new drugs

#### 3.4.1 Protein degradation targeting chimeric compounds (PROTACs)

MYCN‐amplified NB has the worst prognosis, with a 5‐year survival rate of only about 50%. In high‐risk children with NB, MYCN overexpression is associated with histone hyperacetylation. Studies have shown that EP300 is a histone acetylase that regulates MYCN expression. A novel protein degradation targeting chimeric compound (PROTAC) selectively degrading EP300 can lead to rapid apoptosis in NB and represents a potential therapeutic strategy [47].

#### 3.4.2 Nano drug delivery carrier

Osteosarcoma is the most common primary malignant bone tumor in children. Adriamycin is currently one of the first‐line chemotherapy drugs for osteosarcoma, but its toxic side effects such as cardiotoxicity and bone marrow suppression limit its clinical application. A study found that IL‐11Rα receptors were highly expressed on the surface of osteosarcoma tumor cells, and synthesized redox reaction‐sensitive polymer nanoparticles functionalized with IL‐11 ligands to promote intratumoral accumulation of doxorubicin and reduce side effects, which provided a new drug delivery method for clinical chemotherapy of osteosarcoma [48].

## 4 OUTLOOK

The research and application of optimized chemotherapy regimens and new targeted drugs have provided a new avenue for the treatment of children and adolescents with tumors, especially for patients with relapsed/refractory tumors. These new advances in treatment can improve the remission rate, prolong survival time, and solve resistance problems of treatment for pediatric tumor patients. The combination of these new targeted drugs and chemotherapy is a new treatment measure worthy of future trials and clinical advancement, injecting new vitality into the expansion of first‐ and second‐line treatment options and the exploration of new bridging hematopoietic stem cell transplantation options.

## AUTHOR CONTRIBUTIONS

**Jing Tian**: Writing—original draft (Equal). **Jiayu Wang**: Writing—original draft (Equal). **Sidan Li**: Writing—review & editing (Lead).

## CONFLICT OF INTEREST STATEMENT

The authors declare no conflict of interest.

## ETHICS STATEMENT

Not applicable.

## INFORMED CONSENT

Not applicable.

## Data Availability

Not applicable.
